# The Reaction of Oxy Hemoglobin with Nitrite: Mechanism, Antioxidant-Modulated Effect, and Implications for Blood Substitute Evaluation

**DOI:** 10.3390/molecules23020350

**Published:** 2018-02-07

**Authors:** Denisa Hathazi, Florina Scurtu, Cristina Bischin, Augustin Mot, Amr A. A. Attia, Jacob Kongsted, Radu Silaghi-Dumitrescu

**Affiliations:** 1 Department of Chemistry, Faculty of Chemistry and Chemical Engineering, Babeş-Bolyai University, 11 Arany Janos Street, 400028 Cluj-Napoca, Romania; hd1619@chem.ubbcluj.ro (D.H.); florinadeac@chem.ubbcluj.ro (F.S.); cbischin@chem.ubbcluj.ro (C.B.); augustinmot@chem.ubbcluj.ro (A.M.); amrattia@chem.ubbcluj.ro (A.A.A.A.); 2 Department of Physics, Chemistry and Pharmacy, University of Southern Denmark, Campusvej 55, 5230 Odense M, Denmark; kongsted@sdu.dk

**Keywords:** hemoglobin, blood substitutes, nitrite, stopped-flow, peroxynitrate

## Abstract

The autocatalytic reaction between nitrite and the oxy form of globins involves free radicals. For myoglobin (Mb), an initial binding of nitrite to the iron-coordinated oxygen molecule was proposed; the resulting ferrous-peroxynitrate species was not detected, but its decay product, the high-valent ferryl form, was demonstrated in stopped-flow experiments. Reported here are the stopped flow spectra recorded upon mixing oxy Hb (native, as well as chemically-derivatized in the form of several candidates of blood substitutes) with a supraphysiological concentration of nitrite. The data may be fitted to a simple kinetic model involving a transient met-aqua form, in contrast to the ferryl detected in the case of Mb in a similar reaction sequence. These data are in line with a previous observation of a transient accumulation of ferryl Hb under auto-catalytic conditions at much lower concentrations of nitrite (Grubina, R. et al. J. Biol. Chem. 2007, 282, 12916). The simple model for fitting the stopped-flow data leaves a small part of the absorbance changes unaccounted for, unless a fourth species is invoked displaying features similar to the oxy and tentatively assigned as ferrous-peroxynitrate. Density functional theory (DFT) calculations support this latter assignment. The reaction allows for differentiating between the reactivities of various chemically modified hemoglobins, including candidates for blood substitutes. Polymerization of hemoglobin slows the nitrite-induced oxidation, in sharp contrast to oxidative-stress type reactions which are generally accelerated, not inhibited. Sheep hemoglobin is found to be distinctly more resistant to reaction with nitrite compared to bovine Hb, at large nitrite concentrations (stopped-flow experiments directly observing the oxy + nitrite reaction) as well as under auto-catalytic conditions. Copolymerization of Hb with bovine serum albumin (BSA) using glutaraldehyde leads to a distinct increase of the lag time compared to native Hb as well as to any other form of derivatization examined in the present study. The Hb-BSA copolymer also displays a slower initial reaction with nitrite under stopped-flow conditions, compared to native Hb.

## 1. Introduction

Under various stress conditions, the redox chemistry of hemoglobin is susceptible to toxic reactions due to the interaction with oxidative stress agents such as peroxide, yielding free radicals and highly-oxidizing states at the iron (ferryl, Compound II) [1,2,3,4,5]. It has previously been shown that polymerization of hemoglobin, as required when attempting to produce useful candidates for artificial oxygen carriers (“blood substitutes”) can lead to an increase in pro-oxidant reactivity (autooxidation, reaction with hydrogen peroxide, free radical generation), and that this tendency can be reduced when co-polymerizing hemoglobin with antioxidants—of which serum albumin is particularly effective [6,7,8].

The reaction between nitrite and oxy hemoglobin or oxy myoglobin, respectively, has been investigated for almost 150 years, with various reaction pathways or steps proven by the detection of various intermediate species [9,10,11,12,13,14,15,16,17,18,19,20]. Besides negative effects such as methemoglobinemia, nitrite was also shown to act as an endogenous source of nitric oxide, with human and animal studies revealing that nitrite supplementation can have a vasodilating action, thus offering protection against hypoxic pathological conditions [21,22,23,24,25,26,27,28,29].

The oxy + nitrite reaction displays complex kinetics starting with a slow phase (lag) followed by a fast autocatalytic phase involving branching steps and reactive intermediates. Evidence for the implication of hydrogen peroxide, ferryl species, nitrogen dioxide radical and peroxynitrate has been reported for various stages of the reaction [10,11,12,13,14,15,16,17,30,31]. The reaction has been thought to be initiated via one of two ways, not necessarily mutually exclusive or mutually incompatible in the long time scale of the autocatalytic process: (i) direct reaction of nitrite with the iron-bound oxygen and/or (ii) dissociation of molecular oxygen from heme followed by reaction of the heme iron with other ligands, be they nitrite or other small molecules that may result in an aerobic solution of nitrite in the presence of a protein capable of mild catalysis [10,23,32,33,34,35,36,37] such as hemoglobin. For the initial step of the process, binding of nitrite directly to the iron-coordinated oxygen molecule is favored, leading to the formation of an Fe(II)-peroxynitrate adduct, Fe^II^-OONO_2_. Upon the release of nitrate, this would lead to a ferryl species [Fe^IV^ O]^2+^, which has been observed experimentally as such, under longer-scale times (as part of a complex set of competing reactions) as well as under millisecond-timescales (as a result of direct reaction with excess nitrite) [9,11]. The ferryl is known to be reduced by nitrite, thus yielding [Fe^III^-OH_2_] [11,38]. Then, if excess nitrite is used, [Fe^III^-NO_2_] would ensue. Having previously shown that ferryl is clearly detectable in the reaction on oxy Mb and nitrite under stopped-flow millisecond conditions at high nitrite concentrations where the initial oxy+nitrite reaction is directly observable [9], we report here on the distinctly different behavior of hemoglobin in the same reaction—revealing at the same time a potential application as a test for blood substitute candidates in terms of reactivity towards a component of nitrosative stress. Additionally, the stopped-flow data reveals a previously undescribed intermediate, possibly a peroxynitrate adduct formed prior to ferryl. The existence of such an intermediate was previously demonstrated indirectly based on kinetic arguments, though its spectrum was not observed directly [11]. Upon completion of this study, a recent report by Bulow and co-workers has provided additional evidence for the facts that the reaction of oxyHb with nitrite involves metHb and nitrate as final products, and ferryl Hb is an intermediate species of the reaction when working at a low-millimolar concentration level of nitrite, as also initially observed under similar auto-catalytic conditions where, at ~10 s after mixing, a brief accumulation of ferryl was detected [31]. Moreover, they observed that adult human hemoglobin was oxidized more slowly than fetal human hemoglobin with an excess of nitrite [39].

## 2. Results and Discussion

### 2.1. No Significant Accumulation of Ferryl in the OxyHb–Nitrite Reaction at Supraphysiological Nitrite Concentration

**Figure 1:**
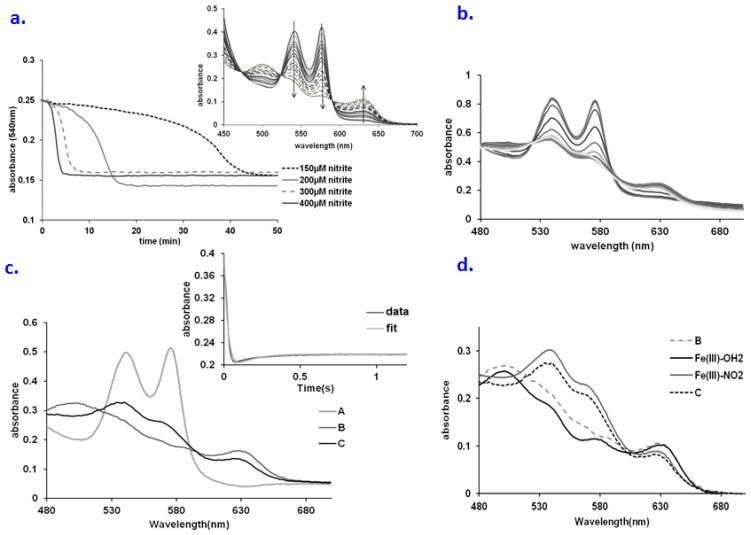
(**a**) Time courses for the nitrite oxidation of hemoglobin in oxygen-saturated phosphate buffer saline (PBS) buffer at various concentrations of nitrite. Conditions: 25 µM Hb, PBS 7.4, room temperature; (**b**) UV-vis spectra collected upon mixing oxyHb (66.6 µM) with NaNO_2_ (660 mM). Conditions: pH 7.4, PBS buffer, aerobic, over a range of 2 s; (**c**) Computed spectra for the species involved in the A → B → C reaction model (A—oxyHb, C—metHb, D—met-nitriteHb). Conditions: 66.6 µM Hb, 0.66 M nitrite, pH 7.4, PBS buffer, aerobic. Inset: fitting at 575 nm trace for the A → B → C kinetic model; (**d**) Overlay of the computed spectra of species B and C with the spectra of various possible intermediates. Conditions: Fe(III)-OH_2_: oxyHb 30µM, pH 7.4; Fe(III)-NO_2_^−^: 30 µM metHb, 400 µM NaNO_2_, pH 7.4 PBS buffer; Fe(III)-OH_2_:oxyHb 30µM, pH 7.4 PBS buffer.

The reaction between nitrite and oxy Hb is influenced by the concentration of reactants in complex manners, especially at lower nitrite concentrations—linked to the fact that several processes are thought to occur beyond the initial event of oxyHb being oxidized to met by nitrite, with NO_2_ as a likely product (the oxy, met and ferryl forms of Hb are all proposed to react with several side-products of initial oxidation events—mainly NO_2_, superoxide, peroxide) [12,13,14,15,16,17,19,20,40]. Figure 1a illustrates that the concentration of nitrite changes the rate of the reaction significantly. Thus, the lag time is directly correlated with the nitrite amount, in the range of 5–50 min. A concentration of 200 µM nitrite was chosen for the ensuing experiments, so that the lag may be clearly observable without excessive lengthening of the experiment. The amount of protein also controls the rate of the process. A lag time is always noted, regardless of the protein concentration. Interestingly, the lag time is inversely correlated with the hemoglobin concentration, in the range of 20–50 min; this may be explained in terms of the excess protein material quenching the free radicals, and thus delaying the onset of oxidation (Supplementary information-Figure S1).

Figure 1b illustrates the UV-vis spectra collected upon mixing of oxy Hb with a very large excess of nitrite—under conditions similar to those previously presented for Mb [9]. As discussed before [9], the reason for using such large concentrations is that under these conditions the initial interaction between Hb and nitrite, which is normally very slow, can be sped up due to the much larger concentrations of reactants—thus increasing the chances of directly observing the reaction product before it would decay via reductive or dissociation processes. The dominant features are those of oxyHb (538–577 nm) and of the met-nitrite adduct (530–575 and 630 nm). The spectra were fitted according to an A → B → C scheme (Figure 1c) where indeed, as in the case of Mb [9], species A is assigned as oxyHb, and C is assigned as the met nitrite adduct (Figure 1d). Surprisingly, in contrast to Mb, where in the similar experiment species B was found to be ferryl, for Hb the spectrum of species B corresponds to a high-spin met Hb (Fe^III^-OH_2_), with the typical absorbance features at 505, 580 and 630 nm (cf. Figure 1c). The decay product of ferryl (including reduction by a number of agents, nitrite included) is indeed known to be a high spin Fe^III^-aqua. In this context, it may be interpreted that Mb and Hb employ the same mechanism for the oxy + nitrite reaction, via a peroxynitrate intermediate, but that the relative reaction rates for ferryl formation and decay are close to each other (as indeed was previously reported for Mb [9]), so that the small structural differences between the two globins (as well as the availability of three neighboring hemes as possible electron donors) [1] can lead to a different behavior, where ferryl can accumulate in higher concentrations in the case of Mb compared with Hb, especially at supraphysiological nitrite concentration. Hogg and co-workers have indeed previously shown that the kinetic behaviour of the Hb–nitrite mixture at physiologically-relevant concentrations can best be reconciled with a mechanism entailing such a peroxynitrate intermediate [11].

**Figure 2:**
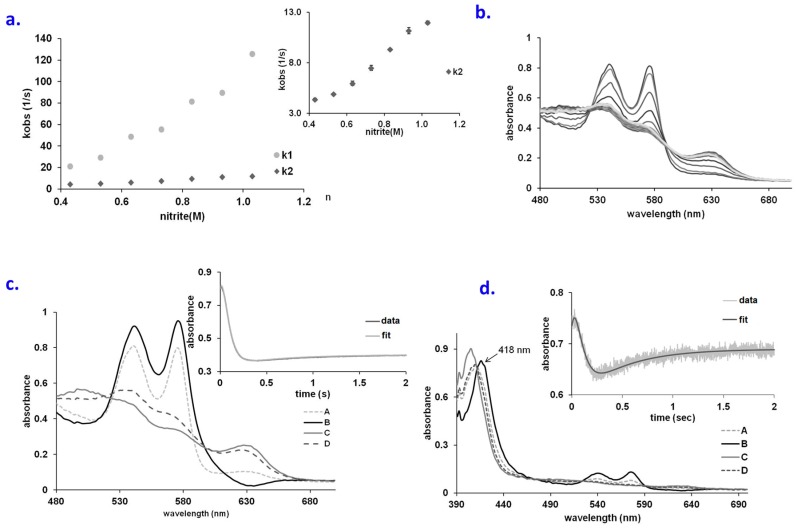
(**a**) Plots of k_1_ and k_2_ vs. NO_2_^−^ concentration for the reaction of oxyHb with NO_2_^−^ at pH 7.4, aerobic; (**b**) UV-vis spectra collected upon mixing oxyHb (66.6 µM) and guanidine (1M) with NaNO_2_ (66 mM). Conditions: pH 7.4, PBS buffer, aerobic, over a range of 2 s; (**c**) Computed spectra for the species involved in the A → B → C → D reaction model (A—oxy Hb, B—Fe(II)-peroxynitrate, C-metHb, D—metnitriteHb). Conditions: 66.6 µM Hb, 1 M guanidine, 66 mM nitrite, pH 7.4, PBS buffer. Inset: fitting at 575 nm trace for the A → B → C → D kinetic model; (**d**) Computed spectra for the species involved in the A → B → C → D reaction model (A—oxy Hb, B—Fe(II)-peroxynitrate, C—metHb, D—met-nitriteHb). Conditions: 6.6 µM Hb, 33 mM nitrite, pH 7.4, PBS buffer, aerobic. Inset: fitting at 575 nm trace for the A → B → C → D kinetic model.

The formation of the met-aqua species B is dependent on the concentration of nitrite with apparent rate constants of 112 M^−1^ s^−1^ for the A → B reaction and 11.5 s^−1^ for the B → C with respect to nitrite at a hemoglobin concentration of 30 μM, (cf. Figure 2a), with a reaction order of 2 for the first reaction and 1 for the second reaction, with respect to nitrite (see also Supplementary information Figures S2–S4). The reaction order for aqua-met formation is consistent with the fact that two nitrite molecules are needed for this reaction: one for generating ferryl from oxy, and one for reducing the ferryl; apparently, these two processes are concerted enough to make the reaction display an order of 2 with respect to nitrite. An alternative interpretation for the non-linearity may also be that the mechanism is even more complicated than proposed/described above (i.e., in addition to involving the concerted formation and decay of ferryl with two nitrite molecules). By contrast, the met + nitrite → met-nitrite (B → C) process displays the expected order 1 for the incoming ligand.

The above-discussed occurrence of an observable met-aqua intermediate instead of an observable ferryl in the reaction of oxy Hb with nitrite as opposed to the ferryl seen with Mb under similar conditions is not altogether unexpected. In Hb, the presence of the three extra subunits and hemes makes for an excess of potential pathways for diffusing the extra oxidizing equivalent in ferryl. Arguably, the comparative instability of the Hb ferryl offers a physiological advantage against oxidative and nitrosative stress agents. On the other hand, under auto-catalytic conditions at much lower nitrite concentrations, a brief accumulation of ferryl Hb in the reaction of oxy Hb with nitrite was indeed reported—though on a much longer time scale (~10 s) compared to the present stopped-flow experiments [31].

### 2.2. An Elusive Minor Component in the Kinetics: The Heme-Peroxynitrate Adduct?

**Figure 3:**
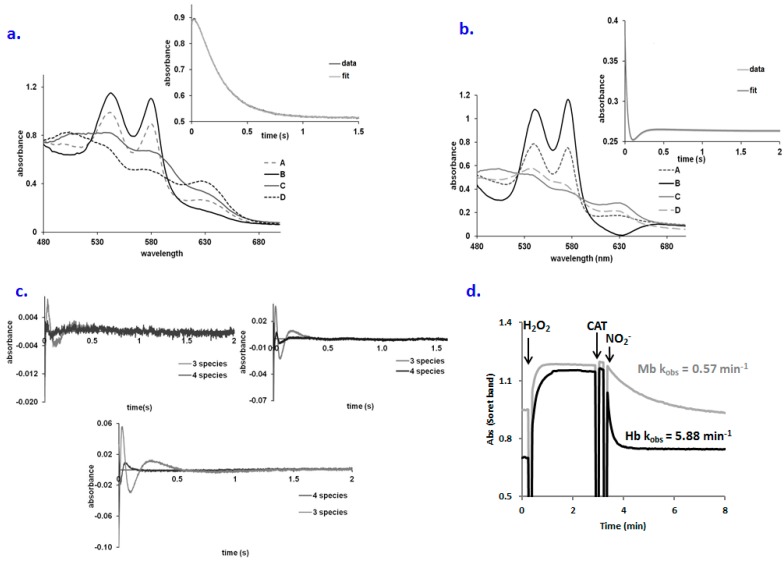
(**a**) Computed spectra for the species involved in the A → B → C → D reaction model for oxyMb + nitrite (A—oxyMb, B—Fe(II)-peroxynitrate, C—metMb, D—met-nitriteMb). Conditions: 75 µM Mb, 0.5 M pH 7.4, PBS buffer, aerobic. Inset: fitting at 575 nm trace for the A → B → C → D kinetic model; (**b**) Computed spectra for the species involved in the A → B → C → D reaction model for oxyMb + nitrite (A—oxyMb, B—Fe(II)-peroxynitrate, C—metMb, D—met-nitriteMb). Conditions: 75 µM Mb, 0.5 M pH 7.4, PBS buffer, aerobic. Inset: fitting at 575 nm trace for the A → B → C → D kinetic model; (**c**) Residual plots of the A → B → C kinetic model vs. A → B → C → D kinetic model for the: left panel: oxy Mb + nitrite, right panel: oxyHb +nitrite, lower panel: oxyHb-guanidine + nitrite. Conditions cf. Materials and Methods; (**d**) Ferryl formation from the met form and its reduction with nitrite for both Mb and Hb (8 µM protein, 100 µM hydrogen peroxide, 0.3 µM catalase (CAT) for excess of peroxide removal, 0.3 mM nitrite, PBS buffer pH 7.4).

The fitting described in Figure 2b–d is imperfect over the starting stages of the reaction, suggesting possible involvement of a fourth species. Acting on the hypothesis that this fourth species may be the missing ferryl (detected so clearly in the Mb experiments), an attempt was made to alter the distal pocket of Hb by increasing its solvent accessibility using controlled concentrations of a denaturing agent. This strategy was previously shown to be efficient in the case of cytochrome *c* [41], where it allowed accumulation of a ferryl species in the reaction with hydrogen peroxide. Figure 2b thus shows UV-vis spectra of guanidine-treated oxy Hb upon mixing with nitrite. The model proposed for this reaction follows A → B → C → D kinetics (Figure 2c and Figures S5–S8), where A oxy, C met-aqua and D met-nitrite. Species B was found to be different from the ones observed until now; it is decidedly different from ferryl, and instead presents a good similarity with the oxy state—with similar positions for the maxima (542 and 575 nm) but higher extinction coefficients—suggesting Fe^II^ with a small ligand [42]; these features are to our knowledge unprecedented, and may be indicative of an (also unprecedented) heme Fe^II^-peroxynitrate adduct. Importantly, the Soret maximum of B was found to be at 418 nm, similar to but shifted compared to oxyHb (Figure 3d). Attempts to enforce a ferryl spectrum for B during the fitting procedure led to a distinctly weaker agreement with experiment (Figure S9, Supplementary information).

Allosteric effects in the case of globins reacting with nitrite have been described [10,31]. The R state of Hb displays a higher reactivity towards nitrite probably due to the lower redox potential or due to the high ligand affinity, which can facilitate the binding of small molecules. This low redox potential favors the distribution of electrons to nitrite and increases the reactivity of this molecule. The reaction of sickle cells with nitrite also provides insights into the allosteric nature of this reaction, thus demonstrating also the role of heme electronics in addition to heme pocket geometry [31]. T-state Hb was shown to have a significantly lower reaction rate with nitrite [32]. To verify that the extra species in Figure 2c is not a manifestation of allostery, the reaction of *met* Hb with nitrite was verified under the same concentration of nitrite as for the oxy Hb + nitrite reaction; no allosteric effects were observed (Supplementary information Figure S10a). More importantly, Figure 3a shows that the reaction of oxy Mb with nitrite may also be fitted to an A → B → C → D scheme—where species B, prior to ferryl, is similar to the one observed with Hb—not only in the presence of guanidine, but also in its absence (Figure 3b). Also, the residual plots of the kinetic data suggest a significantly better fit for the model which involves four species regardless of whether the experiments are done with Hb, Mb or in the presence of guanidine (4c). The concentration interval in which the putative peroxynitrate (B) species was detected is very narrow (33–53 mM nitrite, cf. Figure S6), so the rate constant and the reaction order for the oxyHb-guanidine + nitrite reaction could not be determined. One important aspect that could explain the difference between the behavior of Mb and Hb is the reactivity of the ferryl form with nitrite. Ferryl Hb is reduced much faster by the nitrite as compared with Mb even at low nitrite concentration (Figure 3d) and therefore its accumulation would be expected to be more difficult in the presence of a high nitrite concentration in contrast to Mb.

An alternative interpretation of the data, since we have not detected ferryl in the reaction of oxy Hb with nitrite (though Hogg and co-workers have [11]), would be that ferryl was never formed and that instead the reaction proceeds by initial dissociation of O_2_ from oxy Hb. Such a mechanism was easily discarded for Mb since ferryl was observed [9]. In a putative alternative mechanism, dissociation of O_2_ from oxy Hb would lead to deoxy Hb, which would then react with nitrite to yield ferrous-nitrite (not a detectable species even under stopped-flow conditions [43]) and then ferric-NO. However, neither deoxy nor ferric-NO were detected in our experiments, nor do they offer a reasonable route for accumulation of met-aqua. Instead, Figure 3d does confirm that the reduction of ferryl Hb proceeds much faster than the reduction of ferryl Mb, thus lending support for the interpretation that the ferryl mechanism is at work in both proteins.

The involvement of allosteric effects in the reactions examined in Figure 1 and Figure 2 was also considered. Attempts to fit the stopped-flow data with a concurrent set of two initial reactions (where R and T Hb would react differently) did not improve the results. Bovine Hb, unlike human Hb, is under allosteric control from chloride rather than organic phosphates [8]. Since the experiments in Figure 1 and Figure 2 were conducted in saline phosphate buffer (PBS), hence in the presence of chloride, an attempt was made to examine the oxy + nitrite reaction in the same buffer but now modified to exclude the chloride. At two representative nitrite concentrations (400 and 270 mM), no effect of chloride was noted. Nevertheless, considering the similarity in charge and size between chloride and nitrite, this result is not unexpected. An additional control experiment was performed measuring the affinity of bovine Hb in the presence of nitrite, in the same two buffers as above; here too, no measurable differences were observed (data not shown).

### 2.3. QM/MM and TD-DFT Simulations

Our previously reported [9] DFT calculations on the nitrite + oxy reaction were restricted to the structural (electronic and geometric) analysis of a small model of the heme active center of globins—consisting of the heme (without its lateral substituents) and the imidazole rings of the two nearby histidines (proximal and distal). Negligible barriers were predicted for nitrite binding to the dioxygenic ligand of the iron, as well as for subsequent O–O bond cleavage to liberate nitrate. Figure S14 now shows that even in a more realistic protein environment, such as that of a globin in a QM/MM setting, the energetics of the reaction remain very conducive towards N–O bond formation and ensuing O–O bond cleavage. On the other hand, the same Figure shows that liberation of peroxynitrate from the iron is (just like the liberation of nitrate, and as expected for an anion at a heme center buried in a polypeptide matrix) predicted to be energetically unfavorable.

**Figure 4:**
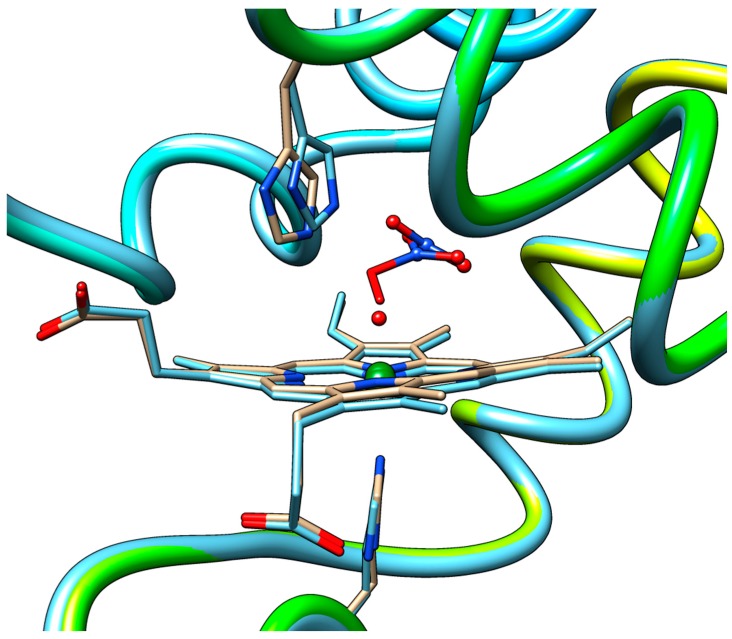
Superimposed QM/MM-optimized geometries of the equilibrium state of the heme-peroxynitrate adduct and that of the state where the O–ONO_2_ bond is totally cleaved.

Figure 4 shows a close-up view of the superposition of the QM/MM-optimized geometries of the equilibrium state of the heme-peroxynitrate adduct and of the state obtained by elongation of the O–O bond via the pathway leading to nitrate liberation up to an O---O distance exceeding the sum of the van der Waals radii. Notably, all of the movement of the NO_3_^−^ moiety up to this point has occurred still in the distal cavity and in fact away from the distal histidine. In fact, the results show that not only the protein backbone retained its equilibrium conformation, but also the side chains of the residues in the vicinity of the heme demonstrate very little movement. This complete rigidity of the protein matrix in the Mb case is already indicating that the energetics of the process will most likely be controlled in a straightforward rigid manner by the interior walls of the distal cavity. The only reasonable way out for an anion from the distal cavity would be via the space opened up by flipping of this His side-chain into the solvent. It is then expected that the mobility of the distal His may control the chances of success in attempts to experimentally detect short-lived caged intermediates such as the heme–peroxynitrate state in globins. On the other hand, the tighter distal cavity of Hb vs. Mb (e.g., with a shorter distance from the Fe to the ceiling of the cavity, Leu29, cf. Figure S15) would force the departing nitrate group to stick for longer times in the vicinity of the heme which may explain why the peroxynitrate intermediate may have been observed directly in Hb, but not in Mb.

The active site was also subjected to TD-DFT calculations, in order to verify any potential support for the hypothesis that a heme-peroxynitrate adduct was indeed detected in the stopped-flow experiments, and that the Soret band of such a species would have higher intensity but similar wavelength to that of the oxy form. As a (second) reference model, the ferryl form of the heme was also computed with the same TD-DFT methodology. As shown in Table 1, the M06-L/6-311g(d,p) TD-DFT data predict the correct trend between the Soret bands of the oxy and ferryl heme: lower energy for the ferryl, and slightly lower extinction coefficients—although the exact wavelength is not accurately predicted for the oxy form. Then, the peroxynitrate adduct is indeed predicted to have the Soret band very close to that of the oxy, and somewhat higher extinction coefficients. Overall, within the limits of the methodology, the TD-DFT data appear to support the conclusion that a heme-peroxynitrate adduct was detected in the stopped-flow experiments, and that its UV-vis spectrum would resemble that of the oxy form.

### 2.4. Behavior of Polymerized Hemoglobin Derivatives

**Figure 5:**
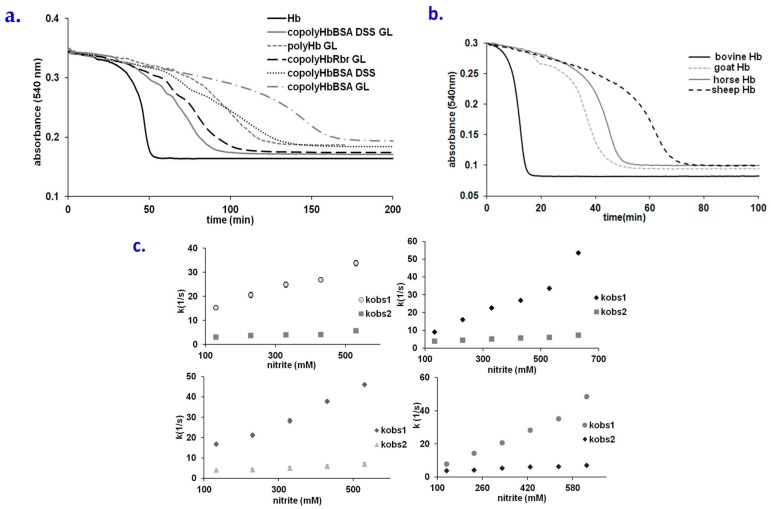
(**a**) Time course for the reaction of native or derivatized Hb (Hb + GL, Hb + BSA + GL, Hb + BSA + DSS, Hb + BSA + DSS + GL, Hb + Rbr + GL cf. Materials and Methods) with nitrite. Conditions: 25µM protein, 200µM nitrite, PBS 7.4, room temperature; (**b**) Time course for the reaction of Hb from different sources (cow, goat, horse, sheep) with nitrite. Conditions: 30 µM protein, 250 µM nitrite, PBS 7.4, room temperature; (**c**) Plots of k_1_ and k_2_ vs. NO_2_^−^ concentration for the reaction of different derivatized oxiHb with NO_2_^−^ at pH 7.4, aerobic; Left panel: up-polyHb, down- copolyHbBSA GL, right: up-copolyHbRbr, down-copolyHbBSA DSS. See also Supplementary Information Tables S1, S3 and S6 and Figures S17–S24.

Hb-based blood substitute candidates have been described extensively, although their use has been limited due to their toxicity issues, with most of them being linked to oxidative stress [8,44,45]. Here, we examine the resistance of such candidates with nitrite, as a model system for nitrosative stress. Figure 5a shows a comparison of how various derivatized globins react with nitrite (raw spectra are shown in Supplementary information, Figure S16). Interestingly, unlike in the autooxidation process, where polymerization with glutaraldehyde (GL) or other bifunctional agents such as dissuccinimidyl suberate (DSS) leads to an increase in the autooxidation rates of hemoglobin [6], these modifications in fact slow down the rate at which Hb is oxidized by low concentrations of nitrite. To our knowledge, this is the first example of a reaction involving nitrosative or oxidative stress agents and hemoglobin, which is *not* accelerated, but rather slowed down, by the polymerization procedures typically applied to Hb with the purpose of creating a blood substitute. Hemoglobin co-polymerized with bovine serum albumin using glutaraldehyde displays the largest lag time, much longer than the version that lacks albumin. By contrast, copolymerization of Hb with a peroxidase (rubrerythrin, Rbr) does not increase the lag time (on the contrary, it decreases it compared to the simple polymer). Hemoglobins from other sources than bovine (horse, goat, sheep) display longer lag times—with sheep Hb displaying the longest. From these points of view, copolymerization with albumin or replacement of bovine Hb with sheep Hb would appear to be reasonable choices when attempting to design a blood substitute with improved resistance to nitrosative stress [6,46,47,48,49].

At large excess concentrations of nitrite (suitable for stopped-flow experiments), the lowest reaction rate was found to be for poly Hb (Table 2)—almost two orders of magnitude lower than for native hemoglobin. The copolyHbBSA GL, copolyHbBSA DSS and copolyHbRbr display higher reaction rates in the stopped-flow experiments, compared to the simple polyHb, but still distinctly lower than native Hb, in agreement with the results seen for the overall process in Figure 5b. Compared to the native Hb, the stoichiometry of the blood substitutes in reaction with nitrite is different—which may be interpreted to be due to the structural changes induced by the polymerization process, limiting the accessibility to the heme.

Hemoglobins from various sources react differently with nitrite, as seen in Figure 5b. Thus, it seems that ovine hemoglobin is the most resistant—the lag phase being three times longer than for bovine Hb. The Hbs in Figure 5c were previously characterized in terms of distance between aromatic aminoacids (electrons that come from exogenous agents are directed towards the heme using Tyr 42) and heme [35,36], This distance is directly correlated with the data in Figure 5c: bovine Hb as the most reactive nitrite-reactive hemoglobin has the shortest distance, in contrast with ovine Hb. At higher concentrations of nitrite, the lowest reactivity was also found for the ovine Hb (Table 3).

A key role in the lag phase for the oxyHb + nitrite reaction is played by hydrogen peroxide, whereas nitrogen dioxide appears only during the propagation phase [20]. In this context, it was suggested that the autocatalytic behavior might be relevant for acute in vivo poisoning, while at physiological nitrite concentrations there are antioxidant systems (small antioxidant molecules, proteins and enzymes) that might scavenge the propagator species [20]. Furthermore, hemoglobin’s free radical reactivity, in reactions related to these and where high-valent iron and free radicals are generated, is of more general analytical importance [50,51,52,53,54,55,56,57,58,59,60,61,62,63,64,65,66]. In this context, experiments where small quantities of different antioxidant molecules were added to the nitrite + oxy reaction were also performed as detailed in the Supporting Information (Figure S25). Thus, uric acid, known for its ability to quench free radicals as well as to reduce Fe(IV) but not Fe(III) hemoglobin [20,35], slows down the process significantly already at physiologically relevant concentrations (Figure S25). Also, a more general antioxidant such as ascorbate (able to also reduce Fe(III) to Fe(II)), present in plasma and in red blood cells at concentrations of 50–250 μM, block the nitrite-induced oxidation of Hb even at concentrations 10 times smaller than the physiological ones—e.g., 5 μM ascorbate (Figure S25). An exogenous antioxidant of polyphenolic nature, caffeic acid, also slows down significantly the nitrite-induced oxidation, suggesting possible utility of this class of compounds as antidotes for nitrite toxicity. *N*-acetyl cysteine, used as a mimic of cysteine-containing peptides such as glutathione, can also slow down the nitrite-induced oxidation—albeit apparently less efficiently than caffeic acid (Figure S25). Last but not least, bovine serum albumin (BSA) also acts efficiently to quench the nitrite-induced oxidation of hemoglobin (Figure S25). This is in line with our previous findings that BSA acts to efficiently reduce the autooxidation and pro-oxidant reactivity of Hb [6,46,47,49]. These findings on antioxidant effects may add to the knowledge to be taken into account when defining the useful concentrations of antioxidants to be added to/with blood substitute preparations in transfusions. The differences in oxy + nitrite kinetics between native and polymerized hemoglobins, as well as those between various animal hemoglobins, can be explained as being due to three factors. First, slightly different solvent accessibility of the heme pocket (either induced by conformational constraints due to polymerization, or natively extant among various animal hemoglobins) may explain the varying rates of nitrite binding. Second, the polymerization, by its effect of increasing molecular weight, will place some of the Hb monomers further away from bulk solvent thereby slowing down the access of nitrite to the respective hemes. Third, the presence of multiple subunits and multiple hemes within the glutaraldehyde-induced Hb aggregates will favor routes whereby the oxidizing equivalents within ferryl and free radicals will dissipate within the Hb aggregate instead of being transferred to solution, thus limiting the autocatalytic effect. The latter interpretation would be in line with the fact that the yield and stability of ferryl Hb have been previously shown to be affected by polymerization [7,65,66].

## 3. Materials and Methods

Native bovine Hb was purified and converted into the oxy form by treatment with dithionite followed by desalting, as previously described [42,67,68]. Bovine serum albumin (BSA, fraction V, from Sigma-Aldrich, Schnelldorf, Germany) was used as provided without further purification. Proteins were manipulated in phosphate buffer saline (PBS). Concentrations are given per heme in the case of Hb and per monomer for the rest of proteins. Other reagents used are as follows: sodium nitrite (NaNO_2_, Reactivul Bucuresti, Bucharest, Romania), L-ascorbic acid (Sigma-Aldrich, Schnelldorf, Germany), uric acid, caffeic acid, *N*-acetyl-l cysteine (Merck, Kenilworth, NJ, USA). Stock solutions of these antioxidants were prepared in water-free DMSO.

The reader is referred to our previous extensive reports on the preparation, purification, chromatographic characterization, oxygen affinity, autooxidation and reactivity towards peroxides for the hemoglobin derivatives employed in the present study, namely, purified bovine hemoglobin (Hb) [36,42,62,63], hemoglobin derivatized with glutaraldehyde with or without bovine serum albumin (polyHb [63,65], copolyHbBSA [7]), hemoglobin co-polymerized with rubrerythrin with or without NROR (copolyHbRbr and Hb-Rbr-NROR) [63] and hemoglobin co-polymerized with disuccinimidil suberate with or without glutaraldehyde and BSA (copolyHbBSA DSS, copolyHbBSA DSS GL [6]. Furthermore, the in vitro (vs. human cell cultures) and in vivo (in rat models) behavior of these samples has also been previously described [46,48,62,63,65,66].

UV-vis spectra were recorded on Agilent 8453 (Agilent, Inc., Santa Clara, CA, USA) and Cary 50 (Varian, Inc., Palo Alto, CA, USA) instruments. Stopped-flow spectra were collected at room temperature on a Biologic SFM-300 system equipped with three syringes and capable of sequential mixing, with a high-speed diode array detector. Stopped-flow data were analyzed within the SPECFIT32 software package (BioLogic Science Instruments, Seyssinet-Pariset, France) using Singular Value Decomposition (SVD) and global multiexponential fitting of the SVD treated data, with the spectra fitted to simple kinetic models using Levenberg–Marquardt or Simplex algorithms.

A computational model based on the X-ray crystal structure of oxy-myoglobin (PDB ID: 2Z6T) [69] was constructed and imported into the Maestro module available in the Schrödinger suite [70]. Peroxynitrate was modeled by the addition of NO_2_ to the oxy ligand already bound to the heme unit. The model was further optimized by adding hydrogen atoms and assigning correct bond orders using the Protein Preparation Wizard [67]. The protonation states of all residues were predicted by the PROPKA module provided in the Protein Preparation Wizard. QM/MM geometry optimizations were performed on the whole system using the Qsite module of the Schrödinger suite [68]. The iron-heme unit along with the peroxynitrate ligand and key residues (His93, His64) were considered in the QM region and optimized with the M06-L DFT functional [71] and the lacvp** basis set. The rest of the enzyme was considered in the MM region and optimized using the OPLS-2005 force field. In addition, QM/MM optimizations of an oxy and oxo model systems were also performed as control structures utilizing the same methodology mentioned above.

**Figure 6:**
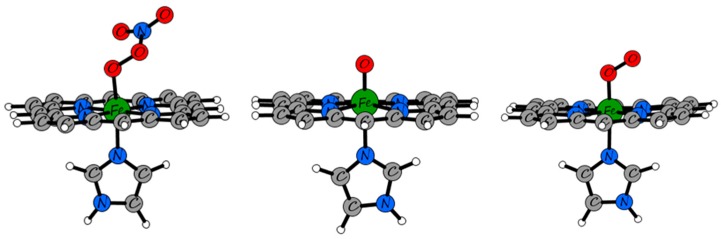
M06-L/6-311g(d,p)-optimized models used in the TD-DFT calculations.

TD-DFT calculations were carried out on simplified models of the active site derived from the QM/MM optimized geometries, i.e., a heme unit without lateral constituents, an axial imidazole and peroxynitrate, oxy and oxo species at the 6th coordination positions. The M06-L density functional and the 6-311g(d,p) basis set were employed in these calculations (Figure 6).

## 4. Conclusions

In myoglobin as well as hemoglobin, stopped-flow data supported by QM/MM and TD-DFT calculations suggest a reaction sequence where the oxy heme reacts with nitrite directly to form a ferrous-peroxynitrate adduct and then ferryl, in line with previous kinetic proposals [11,31]. While only traces of this adduct are seen under native conditions, it accumulates to larger amounts under partially denaturing conditions with guanidine. This intermediate species then generates ferryl via O–O bond cleavage, followed by reduction to met-aqua by excess nitrite, and then by formation of a met-nitrite adduct. Small differences in reactivity between the respective steps led to the unexpected finding that ferryl is clearly accumulated as an intermediate in the case of Mb, whereas met-aqua is the main intermediate accumulated in the case of Hb and ferryl to a much lesser extent. Contrary to oxidative stress reactions, any form of polymerization seems to slow down the reaction of oxy Hb with nitrite. The addition of free albumin, as well as of free small-molecule antioxidants, to the reaction mixture does indeed block the process. Copolymerization of Hb with bovine serum albumin leads to a distinct increase of the lag time under autocatalytic conditions as well as to a slower initial reaction with nitrite under stopped-flow conditions, compared to native Hb. Sheep hemoglobin reacts with nitrite much slower than bovine Hb under stopped-flow as well as under auto-catalytic conditions. These data thus also support the concept that free radicals accumulate during the lag phase of the oxy + nitrite reaction.

## Figures and Tables

**Table 1:** Tabulated wavelengths of the main Soret bands in peroxynitrate, oxy and oxo heme models derived from M06-L/6-311g(d,p) TD-DFT calculations. The excitations with oscillator strengths of at least ~0.1 and wavelengths higher than 350 nm are listed. Frontier molecular orbitals involved in the excitations as well as their energies can be found in the supplementary information (Table S1).

Model	Oscillator Strength	Excitation Energy (nm)
Heme–peroxynitrate	0.374	379
	0.401	376
Heme-oxy	0.200	379
	0.153	368
	0.359	365
	0.249	355
Ferryl	0.113	458
	0.174	424
	0.094	417

**Table 2:** Rate constants and reaction order calculated for different candidates such as blood substitutes for the oxyHb + nitrite reaction. Conditions: 66 µM protein, various concentrations of nitrite, PBS 7.4, room temperature.

	K_1_	n_1_	K_2_	n_2_
Hb	112 M^−1^ s^−1^	2	11.51 s^−1^	1
polyHb	3.4 M^−1^ s^−1^	0.5	6.3 M^−1^ s^−1^	0.4
copolyHbBSA	73 s^−1^	1.1	7.9 M^−1^ s^−1^	0.4
copolyHbRbr	68.8 s^−1^	0.7	9.3 M^−1^ s^−1^	0.5
copolyHbBSA DSS	75.8 s^−1^	1.1	8.3 M^−1^ s^−1^	0.4

**Table 3:** Apparent rate constants of the reaction between different types of hemoglobin and excess nitrite. Conditions: 66 µM oxyHb, 667 mM nitrite, PBS 7.4, room temperature.

Type of Hemoglobin	k_obs1_(1/s)	Stdev1	k_obs2_(1/s)	Stdev2
Bovine Hb	51.53	0.91	5.75	0.21
Goat Hb	58.30	0.85	10.87	0.35
Cabaline Hb	43.93	1.03	11.36	0.50
Ovine Hb	27.02	0.65	8.48	0.23

## Supplementary Materials

The following are available online at http://www.mdpi.com/1420-3049/23/2/350/s1, Figure S1: Time course for the reaction of oxyHb at different concentrations and nitrite, Figure S2: Rate dependencies for the oxy → met transformation, Figure S3. Rate dependencies for the met → met-nitrite transformation, Figure S4: Formation and decay of the species involved in the A → B → C kinetic model for the oxy Hb + nitrite reaction, Figure S5: Overlay of the computed spectra of species B with the spectra of various possible intermediates, Figure S6: Plots of k1, k2and k3 vs. NO_2_^−^ concentration for the reaction of oxyHb-guanidine, Figure S7: UV-vis spectra collected upon mixing oxyHb and guanidine with NaNO_2_, Figure S8: Formation and decay of the species involved in the A → B → C → D kinetic model for the oxy Hb-guanidine + nitrite reaction, Figure S9: Computed spectra for the species involved in the A → B → C → D reaction model for oxy Hb + nitrite with B and C as fixed species, Figure S10: UV-vis spectra collected upon mixing metHb with NaNO_2_ and corresponding computed spectra, Figure S11: Plots of kobs vs. NO_2_^−^ concentration for the reaction of met Hb with NO_2_^−^, Figure S12: Rate dependencies for the metHb → met-nitrite transformation, Figure S13: Plots of kobs vs. met Hb concentration for the reaction of met Hb with NO_2_^−^, Figure S14: QM/MM computed potential energy surfaces for nitrite-dioxygen bond formation and ferrous-peroxynitrate bond cleavage, Table S1: Tabulated energies of frontier MOs involved in the excitations corresponding to the main Soret bands illustrated in Table 1 in the main text, Figure S15: Superimposed crystal structures of myoglobin and the alpha unit of hemoglobin, Figure S16: UV-vis spectra of derivatized globins, Figure S17: Rate dependencies for the oxy polyHb → met transformation, Figure S18: Rate dependencies for the metpolyHb → metnitrite transformation, Figure S19: Rate dependencies for the oxy copolyHbBSA → met transformation, Figure S20: Rate dependencies for the copolyHbBSA → metnitrite transformation, Figure S21: Rate dependencies for the oxy copolyHbRbr → met transformation, Figure S22: Rate dependencies for the met copolyHbRbr → metnitrite transformation, Figure S23: Rate dependencies for the oxy copolyHbBSA DSS → met transformation, Figure S24. Rate dependencies for the met copolyHbBSA DSS → met-nitrite transformation, Figure S25: Influence of urate, ascorbate, caffeate, acetylcysteine and albumin concentration upon the reaction between oxyHb and nitrite.
