# Supplementary material for: The Reaction of Oxy Hemoglobin with Nitrite: Mechanism, Antioxidant-Modulated Effect, and Implications for Blood Substitute Evaluation
Source: Molecules. 2018 Feb 7;23(2):350. doi: 10.3390/molecules23020350 (PMC6017026; doi:10.3390/molecules23020350)
Supplement: Supplementary file 1 [file molecules-23-00350-s001.pdf]

Supplementary information

The Reaction of Oxy Hemoglobin with Nitrite: Mechanism, Antioxidant-Modulated Effect, and Implications for Blood Substitute Evaluation

Denisa Hathazi,[1] Florina Scurtu,[1] Cristina Bischin,[1] Augustin Mot,[1] Amr A. A. Attia,[1,2] Jacob Kongsted,[2] Radu Silaghi-Dumitrescu[1]*

[11]*Department of Chemistry, Faculty of Chemistry and Chemical Engineering, Babeş-Bolyai University, 11 Arany Janos street, Cluj-Napoca 400028, Romania*

[2]*Department of Physics, Chemistry and Pharmacy, University of Southern Denmark, Campusvej 55, 5230 Odense M, Denmark*

**corresponding author; email: rsilaghi@chem.ubbcluj.ro;*

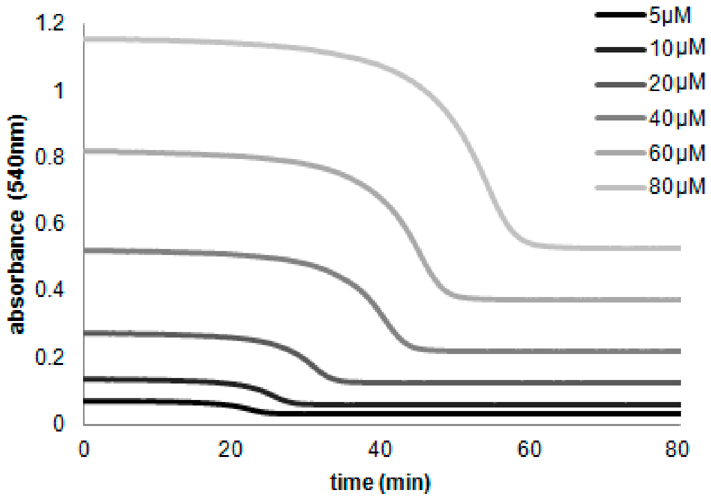

Figure S1. Time course for the reaction of oxyHb (varying concentrations) and nitrite. Conditions: 200µM nitrite, PBS 7.4, room temperature.

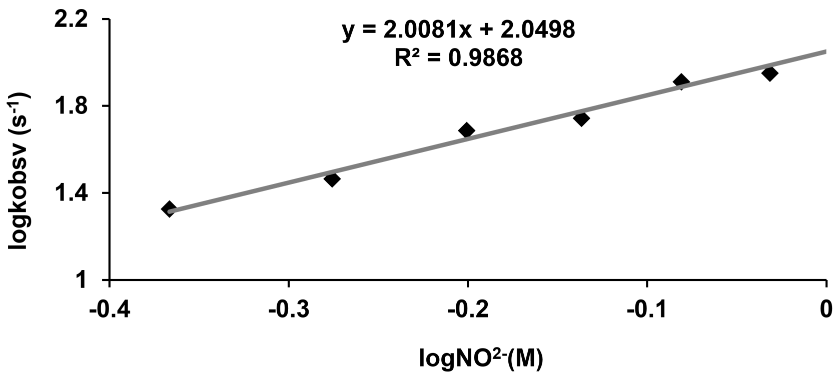

Figure S2. Rate dependencies for the oxy -> met transformation.

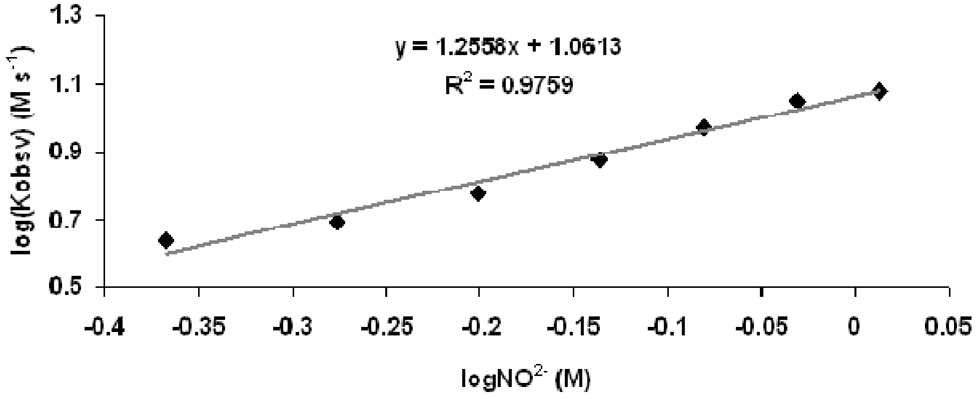

Figure S3. Rate dependencies for the met -> met-nitrite transformation.

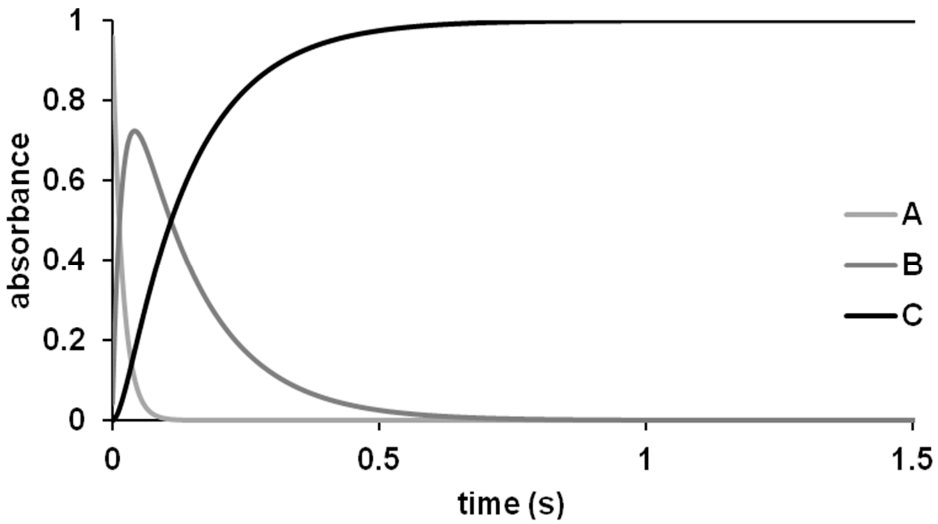

Figure S4. Formation and decay of the species involved in the A→B→C kinetic model for the oxy Hb + nitrite reaction. Conditions: oxyHb (66.6μM) and guanidine (1M) with $NaNO_2$ (66mM), pH 7.4, PBS buffer, aerobic, over a range of 1.5 seconds.

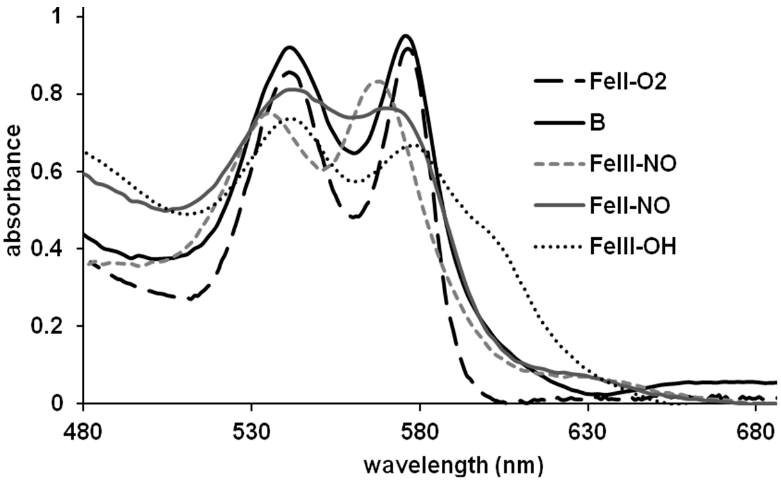

Figure S5. Overlay of the computed spectra of species B with the spectra of various possible intermediates. Conditions: Fe(II)-O_2: oxy Hb 30 µM, PBS buffer, pH 7.4, Fe(III)-OH: oxyHb 30µM, pH 10, 10mM phosphate buffer; Fe(II)-NO: deoxyMb 30 µM, 200 µM NONO-ate, pH 7.4, PBS buffer, anaerobic; Fe(III)-NO: 30 µM Mb, 200 µM 200 µM NONO-ate pH 7.4, PBS buffer, anaerobic.

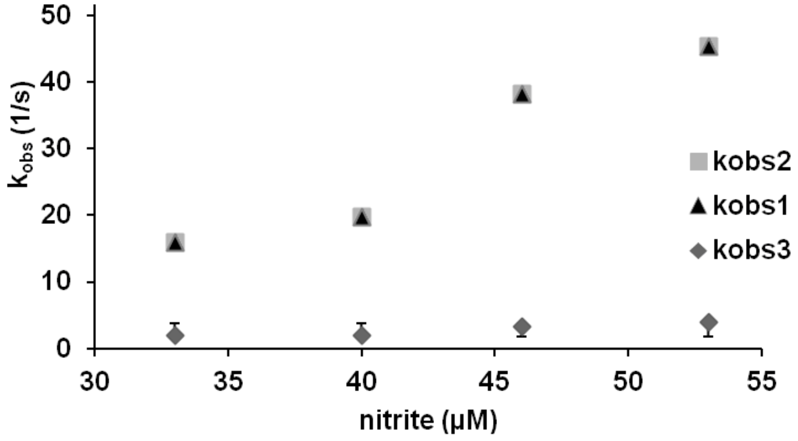

Figure S6. Plots of k_1, $k_2$and k_3 vs. NO_2^- concentration for the reaction of oxyHb-guanidine with NO^-_2 at pH7.4, aerobic.

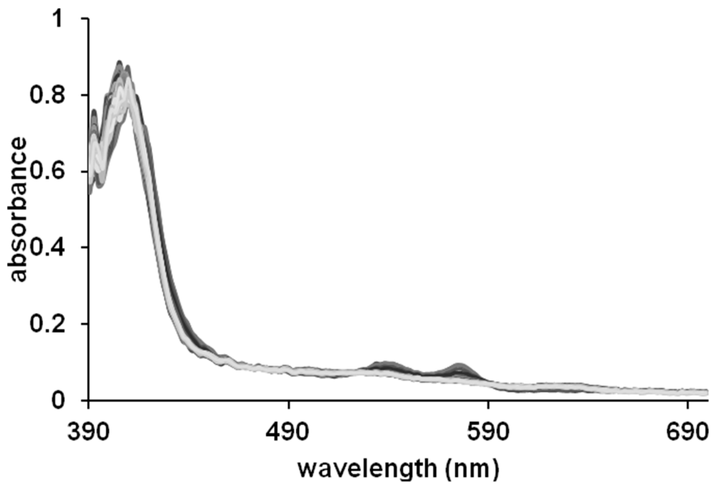

Figure S7. UV-vis spectra collected upon mixing oxyHb (6.6μM) and guanidine (1M) with $NaNO_2$ (66 mM). Conditions: pH 7.4, PBS buffer, aerobic, over a range of 2 seconds.

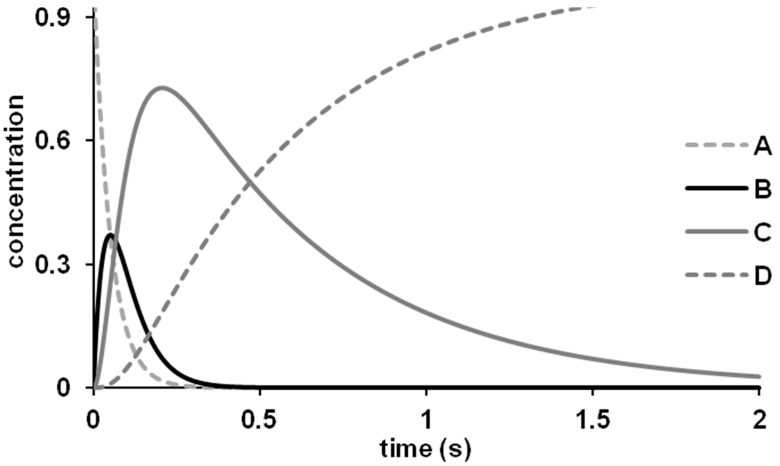

Figure S8. Formation and decay of the species involved in the A→B→C→D kinetic model for the oxy Hb-guanidine + nitrite reaction Conditions: oxyHb (66.6μM) and guanidine (1M) with $NaNO_2$ (66mM), pH 7.4, PBS buffer, aerobic, over a range of 2 seconds.

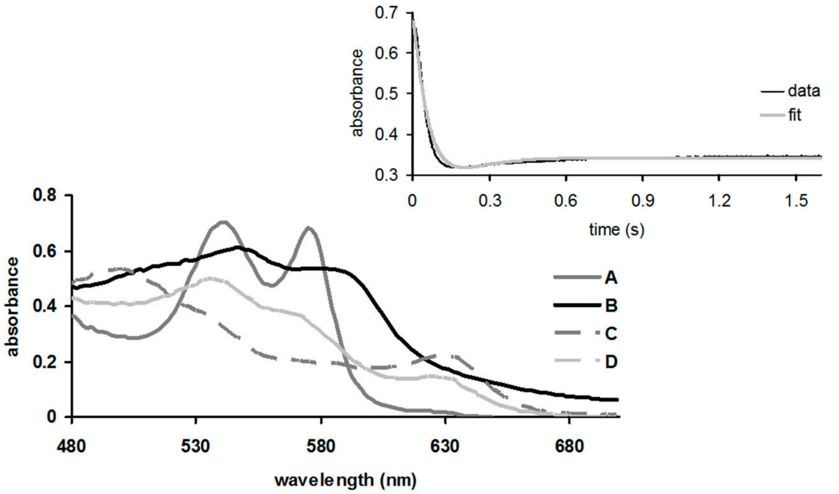

Figure S9. Computed spectra for the species involved in the A→B→C→D reaction model for oxy Hb + nitrite with B and C as a fixed species (A - oxy Hb, B - ferryl, C – met Hb, D - met-nitrite Hb). Conditions: 66 μM Hb, 0.3 M nitrite, pH 7.4. Inset: fitting at 575 nm trace for the A→B→C→D kinetic model.

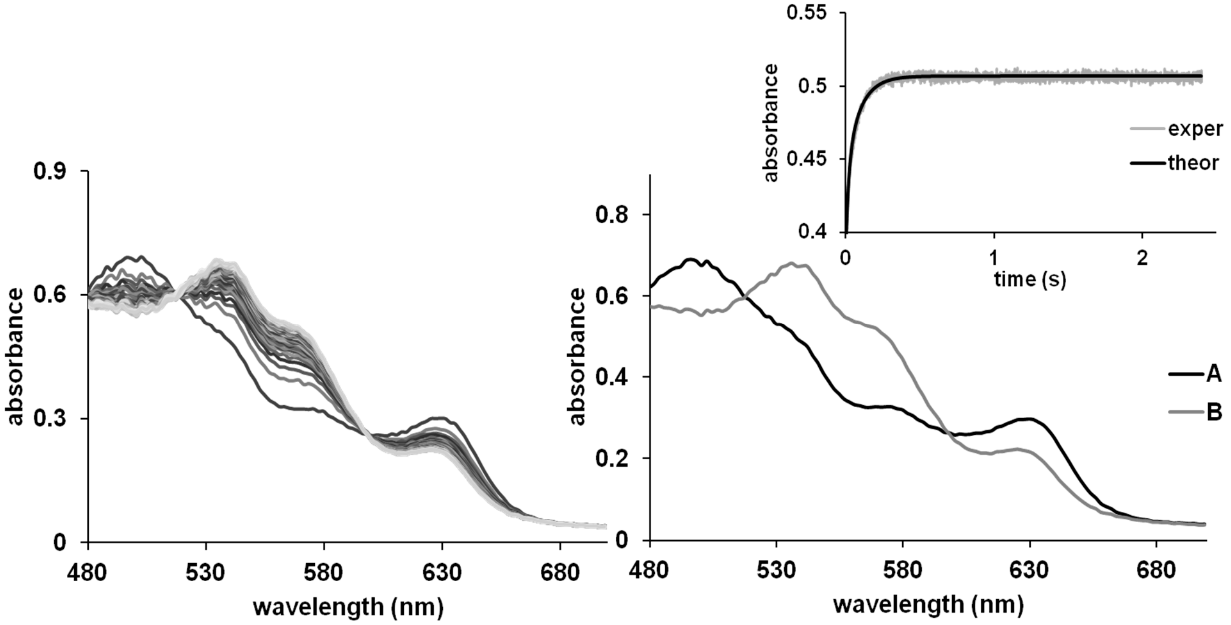

Figure S10. Left panel- UV-vis spectra collected upon mixing metHb (66.6μM) with $NaNO_2$ (0.66 M). Conditions: pH 7.4, PBS buffer, aerobic, over a range of 2.5 seconds; Right panel- Computed spectra for the species involved in the A→B simulated reaction model. Conditions: 66.6 μM metHb, 0.66 M nitrite, pH 7.4, PBS buffer, aerobic; Fitting at 580 nm trace for the A→B→C kinetic model for the met Mb + nitrite reaction.

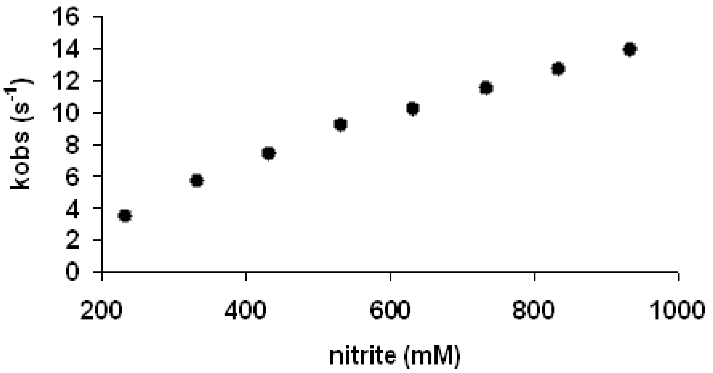

Figure S11.Plots of k_{obs} vs. NO_2^- concentration for the reaction of met Hb with NO^-_2 at pH7.4, aerobic.

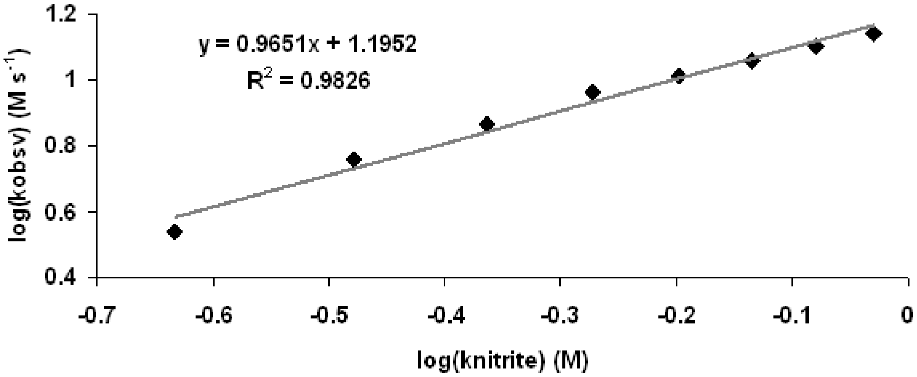

Figure S12. Rate dependencies for the metHb -> met- nitrite transformation.

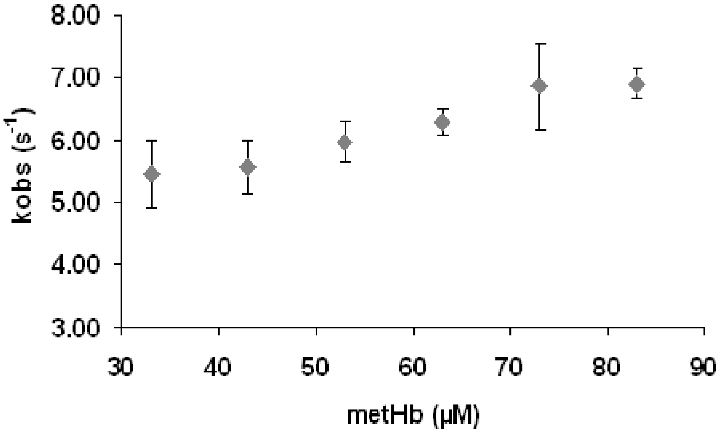

Figure S13. Plots of k_{obs} vs. met Hb concentration for the reaction of met Hb with NO^-_2 at pH 7.4, 0.33 M nitrite, aerobic.

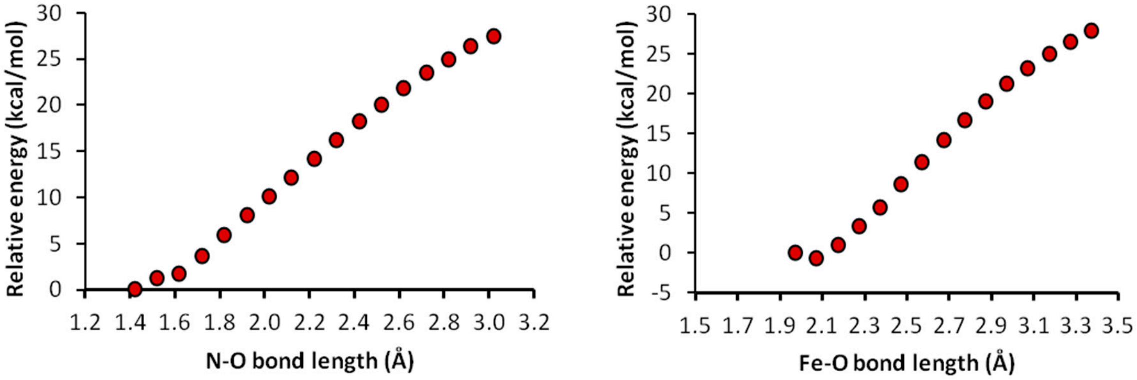

Figure S14. QM/MM computed potential energy surfaces for nitrite-dioxygen (N-O) bond formation (left) and ferrous-peroxynitrate (Fe-O) bond cleavage (right).

Table S1. Tabulated energies of frontier MOs involved in the excitations corresponding to the main Soret bands illustrated in Table 1 in the main text.

**Model**	**MO order**		**MO energy (eV)**	
Heme-peroxynitrate	HOMO -7		-2.56236	
	HOMO -5		-1.46454	
	HOMO -4		-1.34215	
	HOMO -3		-1.12187	
Heme-oxy	HOMO -7		-6.0098	
	HOMO -4		-4.71367	
	HOMO -3		-4.66246	
Heme-oxo	αHOMO -4		2.141129	
		βHOMO -3		2.139877
	αHOMO -1	βHOMO -1	2.452836	2.527558

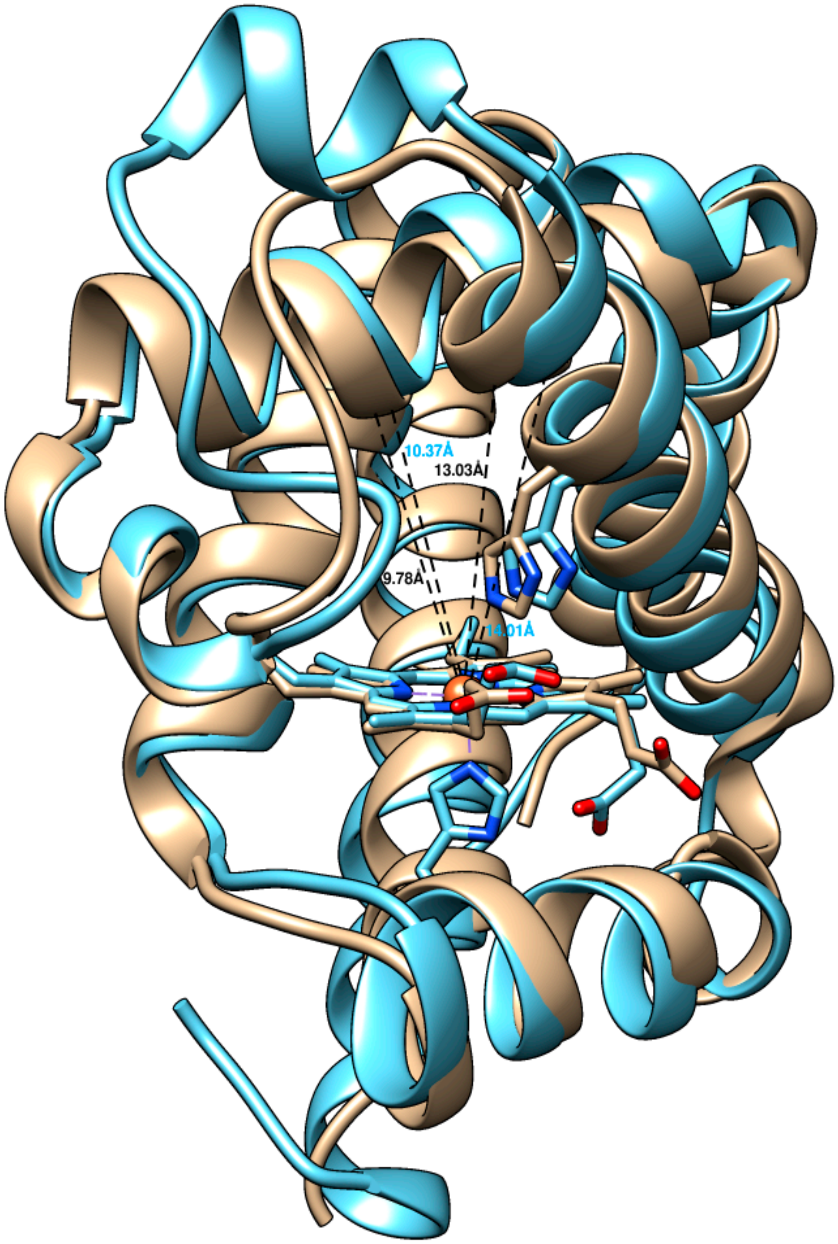

Figure S15. Superimposed crystal structures of myoglobin (light blue) and the alpha unit of hemoglobin (grey). Distances in light blue correspond to those of myoglobin while those in black correspond to hemoglobin.

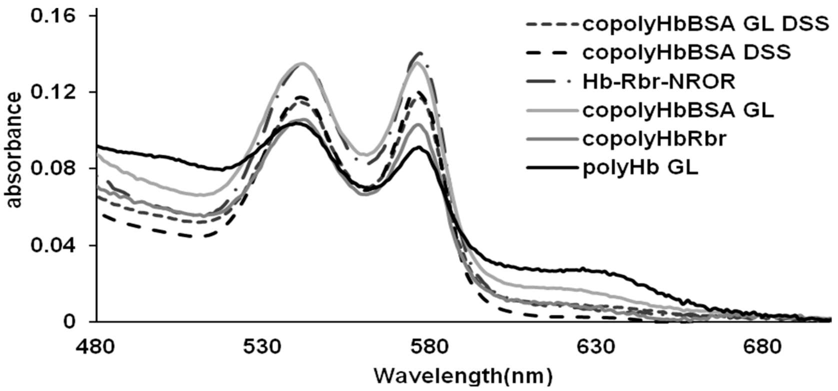

Figure S16. UV-vis spectra of derivatized globins. Conditions: 20 µM protein, PBS pH 7.4.

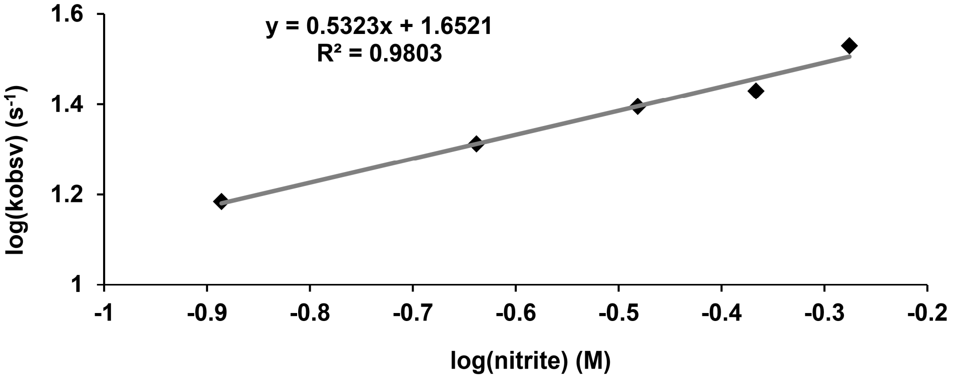

Figure S17. Rate dependencies for the oxy polyHb -> met transformation.

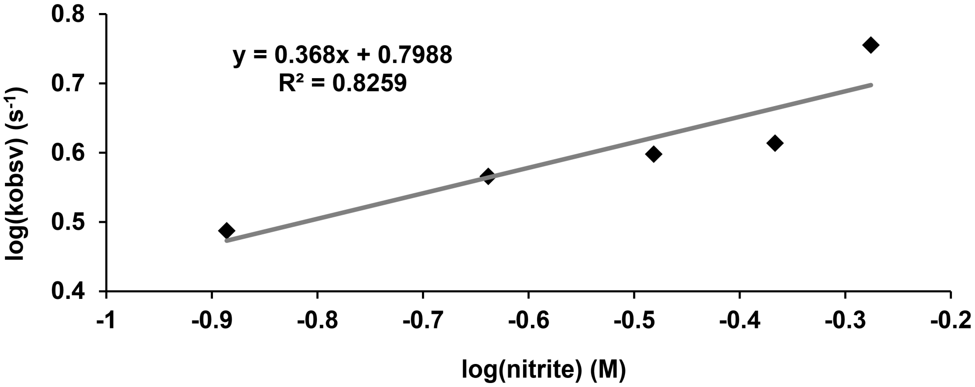

Figure S18. Rate dependencies for the metpolyHb ->metnitrite transformation.

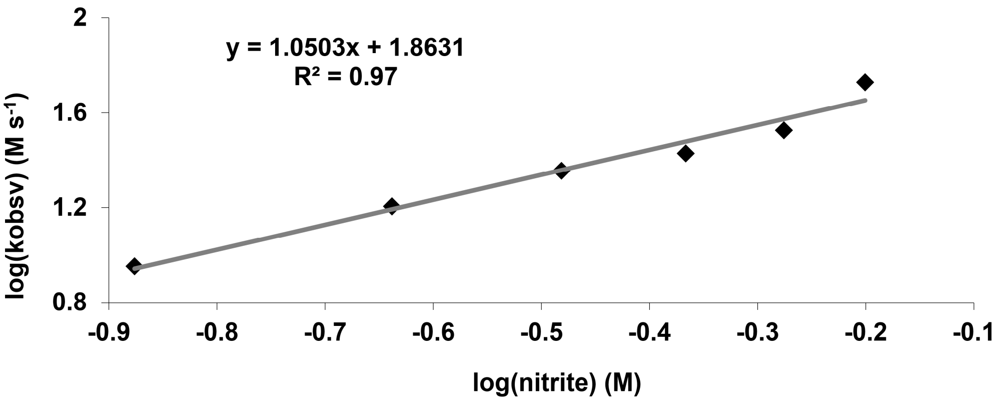

Figure S19. Rate dependencies for the oxy copolyHbBSA -> met transformation.

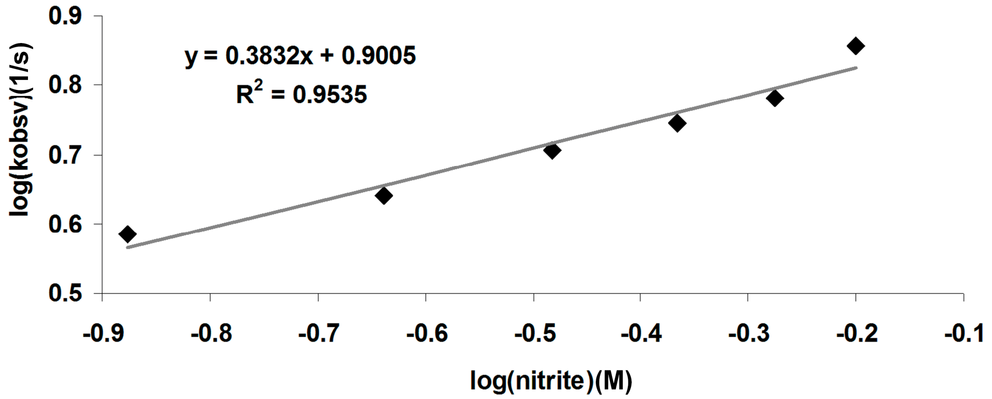

FigureS20.Rate dependencies for the copolyHbBSA ->metnitrite transformation.

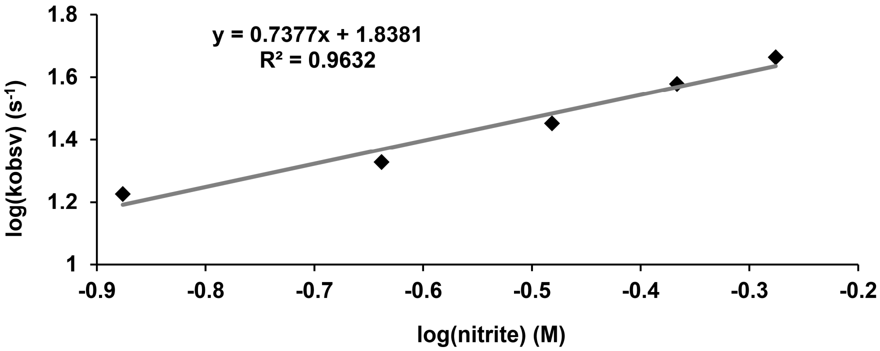

Figure S21. Rate dependencies for the oxy copolyHbRbr -> met transformation.

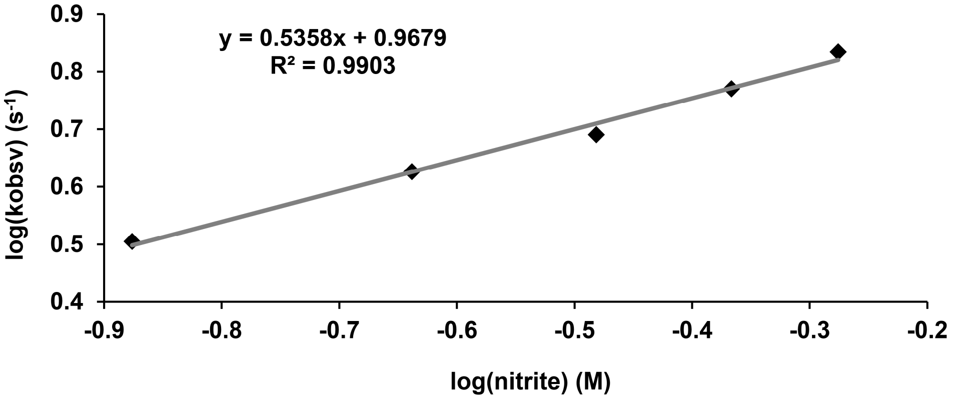

Figure S22. Rate dependencies for the met copolyHbRbr ->metnitrite transformation.

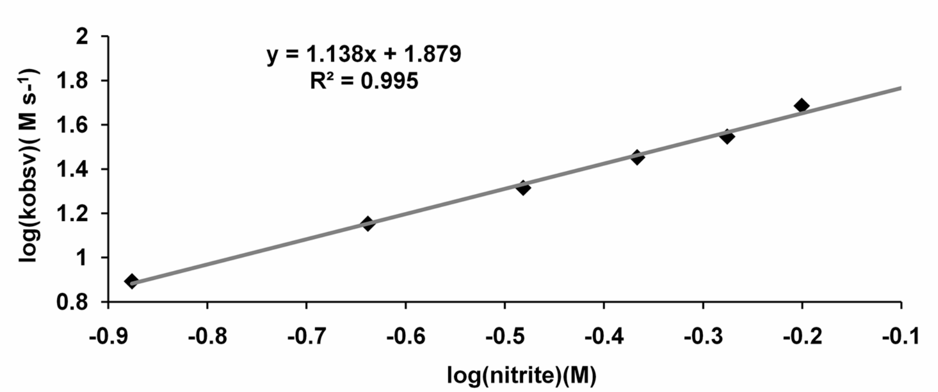

Figure S23. Rate dependencies for the oxy copolyHbBSA DSS -> met transformation.

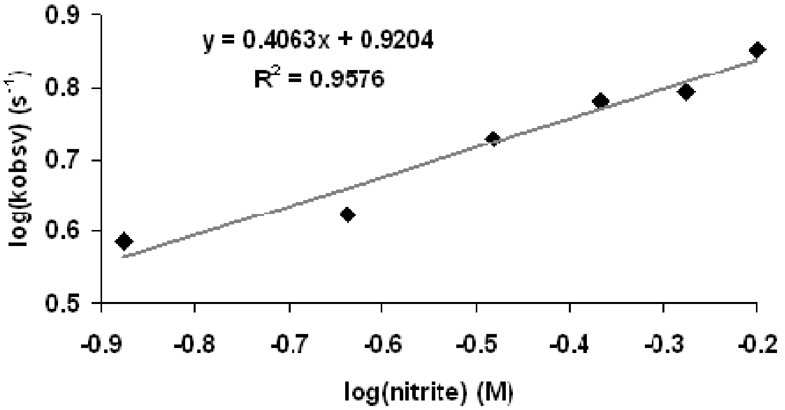

Figure S24. Rate dependencies for the met copolyHbBSA DSS -> met-nitrite transformation

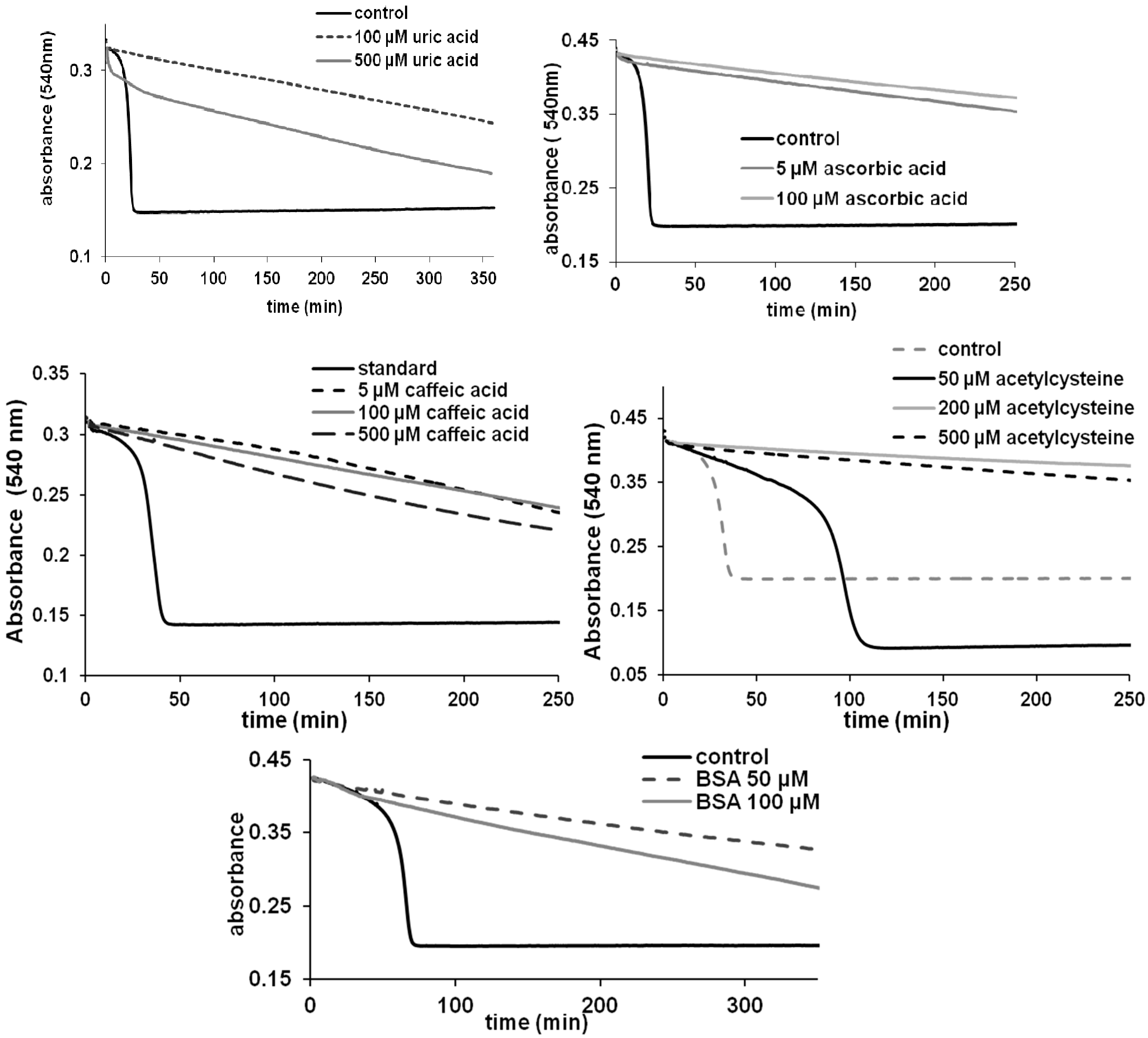

Figure S25. Influence of urate, ascorbate, caffeate, acetylcysteine and albumin concentration upon the reaction between oxyHb and nitrite. Conditions: 25 µM oxyHb, 200 µM nitrite, PBS pH7.4, room temperature; Right: Influence of acetylcysteine concentration upon on the reaction between oxyHb and nitrite. Conditions: 25 µM oxyHb, 200 µM nitrite, PBS pH7.4, room temperature.
